# For your eyes only: effect of confederate’s eye level on reach-to-grasp action

**DOI:** 10.3389/fpsyg.2014.01407

**Published:** 2014-12-04

**Authors:** François Quesque, Yann Coello

**Affiliations:** Psychology Department, Unité de Recherche en Sciences Cognitives et Affectives, Charles de Gaulle-Lille 3 University – University of Lille Nord de FranceVilleneuve d’Ascq, France

**Keywords:** perception, motor action, social intention, eye level, kinematics

## Abstract

Previous studies have shown that the spatio-temporal parameters of reach-to-grasp movement are influenced by the social context in which the motor action is performed. In particular, when interacting with a confederate, movements are slower, with longer initiation times and more ample trajectories, which has been interpreted as implicit communicative information emerging through voluntary movement to catch the partner’s attention and optimize cooperation (Quesque et al., 2013). Because gaze is a crucial component of social interactions, the present study evaluated the role of a confederate’s eye level on the social modulation of trajectory curvature. An actor and a partner facing each other took part in a cooperative task consisting, for one of them, of grasping and moving a wooden dowel under time constraints. Before this *Main action*, the actor performed a *Preparatory action*, which consisted of placing the wooden dowel on a central marking. The partner’s eye level was unnoticeably varied using an adjustable seat that matched or was higher than the actor’s seat. Our data confirmed the previous effects of social intention on motor responses. Furthermore, we observed an effect of the partner’s eye level on the *Preparatory action*, leading the actors to exaggerate unconsciously the trajectory curvature in relation to their partner’s eye level. No interaction was found between the actor’s social intention and their partner’s eye level. These results suggest that other bodies are implicitly taken into account when a reach-to-grasp movement is produced in a social context.

## INTRODUCTION

Humans live in social groups and spend much time engaging in cooperative actions (Richerson and Boyd, 1998) or interpreting observed behaviors (Barresi and Moore, 1996), even when there is no clear intention to interact with conspecifics (Frith and Frith, 2006). Motor actions have the special feature of being influenced by both the goal pursued (Marteniuk et al., 1987; Ansuini et al., 2006, 2008; Naish et al., 2013) and the social context in which they are performed (Ferri et al., 2011; Gianelli et al., 2011; Innocenti et al., 2012; Quesque et al., 2013; Scorolli et al., 2014). As a consequence, observers can detect, from kinematic variations in motor performances, the goal of an ongoing action before it is entirely executed (Orliaguet et al., 1997; Elsner et al., 2012; Stapel et al., 2012; Lewkowicz et al., 2013) and even the actor’s social intention (Sebanz and Shiffrar, 2009; Manera et al., 2011; Sartori et al., 2011). For instance, Manera et al. (2011) showed that observers could readily categorize from movement information whether an object was grasped to perform an individual action or with the intention of socially cooperating. In line with this specific sensitivity to social cues borne by action, recent neuroimaging studies highlighted the capacity of the brain to discriminate very rapidly from the optic flow information relating to human bodies (see de Gelder et al., 2010 for a review) and bodily expressions (see Blake and Shiffrar, 2007 for a review). It has been suggested that the implicit process of socially relevant motor features could optimize cooperation between agents and contribute to the selection of adapted responses depending on the social demands (Gallagher, 2008).

The role of sensorimotor cues in social interactions is a particular aspect of human communication that originates from the very early motor experiences that infants share with their parents (Brand et al., 2002; Brand and Shallcross, 2008). The so-called “motionese” strategy reflects the fact that parents exaggerate their movements when addressing their children. Although less accentuated in later life, this effect does not seem restricted to childhood since changes in kinematics have also been observed when communication occurs between adults in pointing (Cleret de Langavant et al., 2011) and grasping tasks (Sartori et al., 2009). In the latter experiment, participants were asked to reach, grasp, and lift colored spheres for an individual or cooperative purpose requiring an observer to decode a message from the alternation of colors via a simplified Morse code. Although the goal of the motor action was identical in the two conditions for the actor, the reach-to-grasp movements were performed differently when there was a social communication constraint. More precisely, the reaching movements were slower with less straight trajectories in the communicative than in the non-communicative condition. Thus, it appears that when endorsing social intention – that is, when other actors are crucial elements for satisfying the intended goals (Ciaramidaro et al., 2007) – humans tend to modify the kinematics of their motor behaviors, even when there is no explicit instruction to communicate. In agreement with this, when actors move an object to allow a partner (rather than themselves) to perform a goal-directed action, they move and place the objet using a more curved trajectory (Becchio et al., 2008b; Quesque et al., 2013) and a longer movement initiation time (Quesque et al., 2013). Such an increase in movement amplitude has been interpreted as an implicit strategy to catch the partner’s attention and communicate social intention (Quesque et al., 2013); the movements being performed with a higher amplitude due to the partner’s eye level representing a social target that influences the implementation of goal-directed action.

Supporting the assumption of an influence of eye level on cooperative tasks, several studies have pointed out the predominant role of gaze in human social interactions (Argyle and Cook, 1976; Kleinke, 1986; Langton et al., 2000; Becchio et al., 2008a) from the early days of life (Farroni et al., 2002). In comparison with other primate species, humans have especially visible eyes (Kobayashi and Kohshima, 1997) which renders their gaze direction much more salient, thus facilitating cooperative behaviors and joint actions. In studying how social context affects movement kinematics, recent research has led to the conclusion that the appropriate direction of a partner’s gaze is a prerequisite to effective social interactions (Ferri et al., 2011; Innocenti et al., 2012; Scorolli et al., 2014).

In this context, the present study aimed to evaluate the effect of a partner’s eye level on the execution of individual or cooperative voluntary reach-to-grasp movements. If hand elevation when performing an action in a social context is influenced by the height of a partner’s eyes, as suggested by previous studies, hand trajectories would be expected to be higher when a motor action was performed in the presence of a partner taking a higher seated position. Furthermore, this study investigated whether the effect of eye level on the spatio-temporal parameters of motor responses depends on the communicative context, i.e., when a social intention is endorsed, or if it depends on a more implicit influence occurring even in the absence of any social interaction (e.g., Bateson et al., 2006; Ernest-Jones et al., 2011).

## MATERIALS AND METHODS

### PARTICIPANTS

Twenty-one healthy, right-handed (as determined by the Edinburgh Handedness Inventory, Oldfield, 1971) adults (mean age = 21.05 years, SD = 1.96 years, four males) were tested. They had no prior knowledge about the scientific aim of the study and provided their written informed consent before participating. The protocol followed the general ethics rules defined by the local ethics committee and was in accordance with the principles of the Declaration of Helsinki (World Medical Organization, 1996). The experimenter (the first author of this paper) was a 24-year-old man who played the role of the social partner for all participants.

### APPARATUS AND STIMULI

**FIGURE 1:**
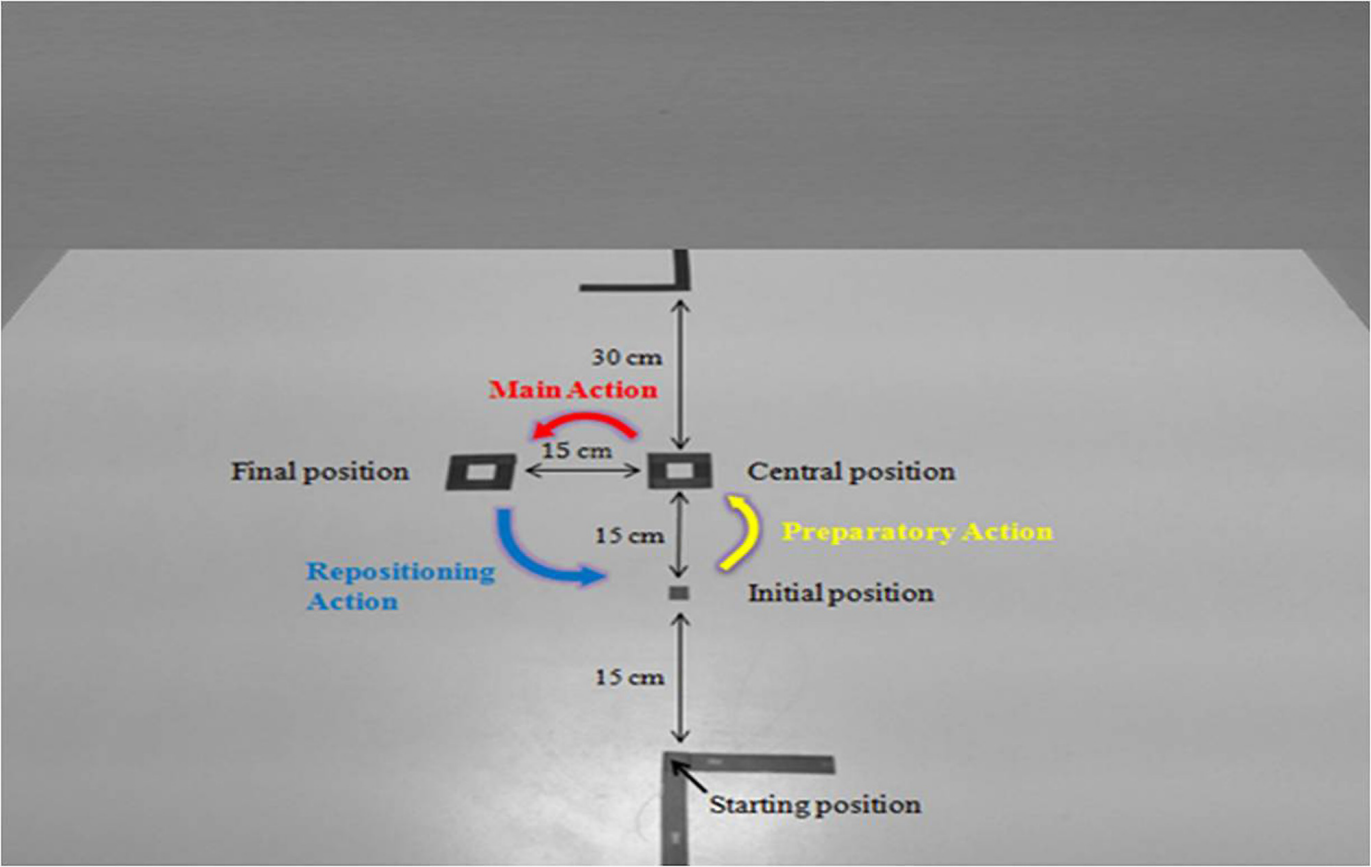
**Top view picture of the experimental setup with the initial, central and final positions, the starting position of the participants, and an illustration of the *Preparatory*, *Main,* and *Repositioning actions***.

Participants and the partner sat on either side of a table (120 cm × 80 cm), facing each other. 2 cm × 2 cm black markings on the table indicated three specific locations, which will be hereinafter referred to as the initial, central and final positions. In addition, the starting positions used for the right hand of the participants and the partner were indicated by black markings located at each edge of the table. The object to be manipulated was a wooden dowel (diameter 2 cm, length 4 cm), which was to be moved from one spatial landmark to the next following a defined sequence, each movement in the sequence being triggered by an auditory cue (see **Figure 1**).

### PROCEDURE

The task for the participants was to reach and grasp the wooden dowel using their thumb and index finger and move it from one position to the next in a sequence of three successive actions. Before performing each action, both the participants and the partner were requested to remain stationary with their thumb and index finger pinched together and resting upon the starting position. Each sequence started with the wooden dowel placed at the initial position. The first action was the *Preparatory action,* which consisted of moving the wooden dowel from the initial to the central position with no specific time constraints. The second action was the *Main action,* in which the wooden dowel was moved horizontally from the central to the final position as fast as possible. The third action was the *Repositioning action,* in which the wooden dowel was moved from the final to the initial position with no specific time constraints, thus setting up for the next sequence. Time constraints were thus only applied to the *Main action*, in which the velocity of the participant’s wrist had to be more than 80% of the maximum reachable velocity (computed from the peak velocity recorded in a previous practice session, see below and Quesque et al., 2013 for a detailed description). Each movement was triggered by a specific auditory cue, always broadcast in the same order (cue 1 initiated the *Preparatory action*; cue 2 the *Main action;* cue 3 the *Repositioning action)*. Thus, participants and the partner had their right hand on the starting positions before initiating any of the movements in the sequence, while their left hand remained in their lap. When the participant or the partner was acting, the other person had to keep motionless. Furthermore, participants were not allowed to communicate and were asked to fix their gaze on the table during the course of the experiment in all sessions. In order to prevent participants from anticipating the time of movement initiation, between-sequences intervals varied randomly between 3 and 3.5 s. In addition, the interval between the first and second auditory cue was varied randomly between 3.5 and 4 s while the interval between the second and third auditory cue was fixed at 2 s in order to provide feedback on the participant’s performance immediately after they had completed the *Main action*.

Participants performed four successive sessions of 25 sequences of action. In these sessions, the *Main action* was carried out by either the participant or the partner (block trials), with the seat of the partner being either at the same height as or higher than that of the participant (block trials). The eye level of the partner was manipulated using an adjustable seat, which was either at the same height as that of the participant (0 cm condition) or 5 cm higher (5 cm condition, counterbalanced order). In order to minimize the risk that participants detected this manipulation, the two height conditions were performed on different days separated by 1 week and the height of the seat was adjusted before the arrival of the participants. In each of these conditions, the participants and the partner performed the *Main action* in two block sessions presented in a counterbalanced order on the same day. Then, depending on the session, when performing the *Preparatory action*, participants could place the wooden dowel for themselves (personal intention) or for their partner (social intention).

### PRACTICE SESSIONS

Before the experimental session started, all participants underwent two practice blocks, each containing 15 sequences of action. The first practice block was done to obtain an estimate of the maximum speed at which participants could grasp the wooden dowel from the central position and place it on the final position. An adjustment procedure similar to the one used in Quesque et al. (2013) was used. The second practice block was done to check that instructions were understood and that the different auditory cues were accurately identified and the appropriate motor responses provided.

### DATA RECORDING AND ANALYSIS

Participants’ motor performances were recorded using Qualisys 4 Oqus infrared cameras (Qualisys AB, Gothenburg, Sweden). Infrared reflective markers were placed on the forefinger (base and tip), thumb (tip) and wrist (scaphoid) of the right hand of participants. An additional marker was placed on the wooden dowel. Cameras were calibrated before each session, enabling the system to reach SD accuracies of less than 0.2 mm, at a 200 Hz sampling rate. Only the *Preparatory action* data were analyzed, because the social influence on motor performances can be estimated only from this action. The *Preparatory action* was characterized by a reaching phase (reach-to-grasp action) and a transport phase (moving the wooden dowel from the initial to the central position). The focus was on movement parameters that are known to be affected by social intentionality, namely reaction time, movement time, peak wrist velocity, and height of the trajectory in the reaching and transport phases (Becchio et al., 2008b; Quesque et al., 2013). Reaction time, movement time and trajectory elevation were computed from the 3D coordinates of the reflective marker placed on the wrist of participants. Temporal and kinematic parameters of the (x,y,z) coordinates of the wrist marker were computed from tangential velocity profiles after filtering the data using a second-order Butterworth dual pass filter (cutoff frequency: 15 Hz). Movement onset was defined as when the first velocity value reached 20 mm s^-1^. Movement end was defined as the time the velocity profile reached the minimum value following the peak velocity of the transport phase. Reaction time corresponded to the time separating the *Preparatory action* auditory cue from movement onset. Movement time corresponded to the time separating movement onset from movement end. Peak wrist velocity corresponded to the maximum velocity reached by the wrist during the grasping and transport phase, respectively. The maximum height of trajectory was defined as the maximum z coordinate of the wrist measured in the grasping and transport phases.

Concerning reaction time, a 2 (Intention: Social vs. Personal) × 2 (Partner’s eye level: 0 cm vs. 5 cm condition) ANOVA was conducted. Concerning movement time and kinematic parameters analysis, 2 (Intention: Social vs. Personal) × 2 (Partner’s eye level: 0 cm vs. 5 cm condition) × 2 (Movement phase: Grasping vs. Transport) ANOVAs were conducted, all variables being associated with within-participants measures. The significance level was set at 0.05 and the problem of multiple comparisons was corrected using the Bonferroni method.

## RESULTS

Trials were excluded from the data analysis when a participant responded erroneously, when the marker was not correctly recorded during the movement, or when the reaction time was shorter than 200 ms or longer than 2.5 SDs from the median (Leys et al., 2013) computed from the *Preparatory actions*. 5.2% of the trials, homogenously distributed across the conditions, were thus excluded.

### REACTION TIME

**FIGURE 2:**
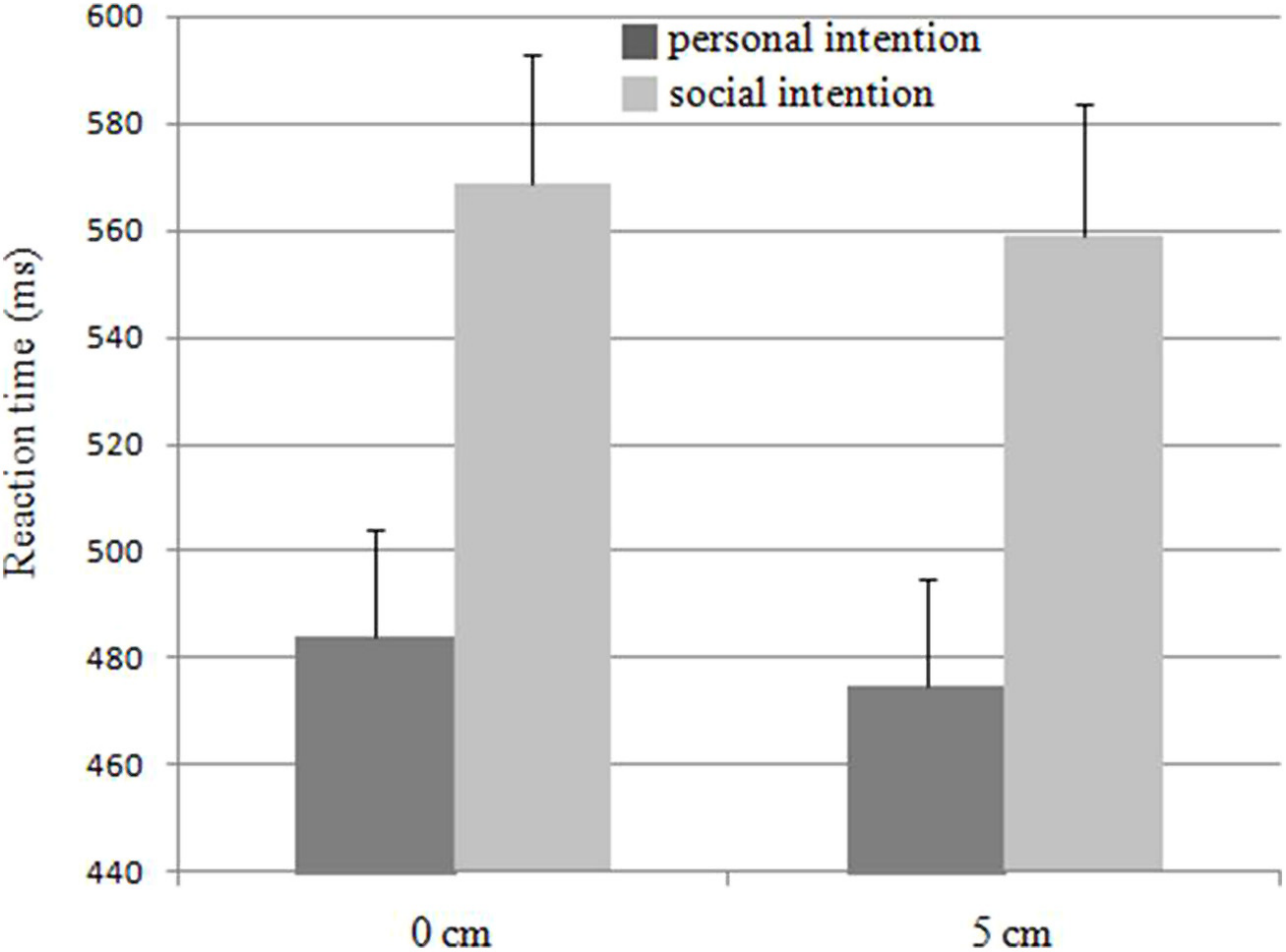
**Reaction time as a function of social intention and partner’s eye level.** Error bars represent the SE.

Reaction time was influenced by social intention [*F*(1,20) = 50.69, *p* < 0.001, $\eta_{p}^{2}$ = 0.72]. Participants showed a longer reaction time when they placed the wooden dowel for the partner (564 ms) than for themselves (480 ms). However, no effect of partner’s eye level on reaction time [*F*(1,20) = 0.62, ns] was found, and there was no interaction between the two factors [*F*(1,20) = 0.99, ns; see **Figure 2**].

### MOVEMENT TIME

**FIGURE 3:**
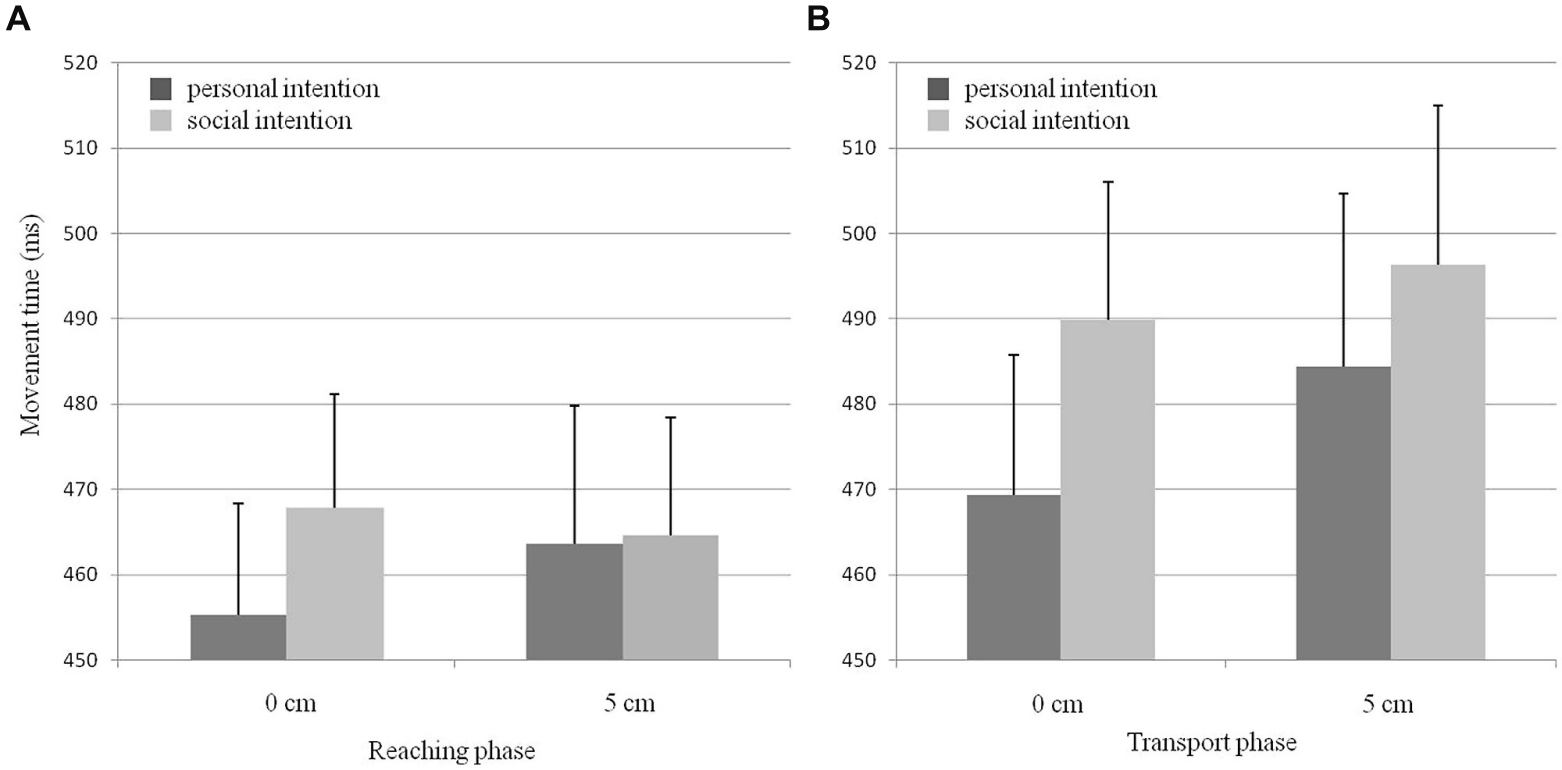
**Movement duration as a function of social intention and partner’s eye level for both (A) the reaching and (B) the transport phases of the *Preparatory action*.** Error bars represent the SE.

Movement time was influenced by social intention [*F*(1,20) = 5.49, *p* < 0.05, $\eta_{p}^{2}$ = 0.22], increasing when a social (480 ms) rather than a personal (468 ms) intention was endorsed. Furthermore, movement time increased in the transport phase (485 ms) compared to the grasping phase [463 ms, *F*(1,20) = 7.15, *p* < 0.05, $\eta_{p}^{2}$ = 0.26]. However, no effect of partner’s eye level on movement time [*F*(1,20) = 1.38, ns] was found, and there was no interaction between the three factors [social intention/movement phase: *F*(1,20) = 2.5, social intention/eye level: *F*(1,20) = 1.71, movement phase/eye level: *F*(1,20) = 2.25, social intention/movement phase/eye level: *F*(1,20) = 0.13, all ns; see **Figure 3**].

### WRIST ELEVATION

**FIGURE 4:**
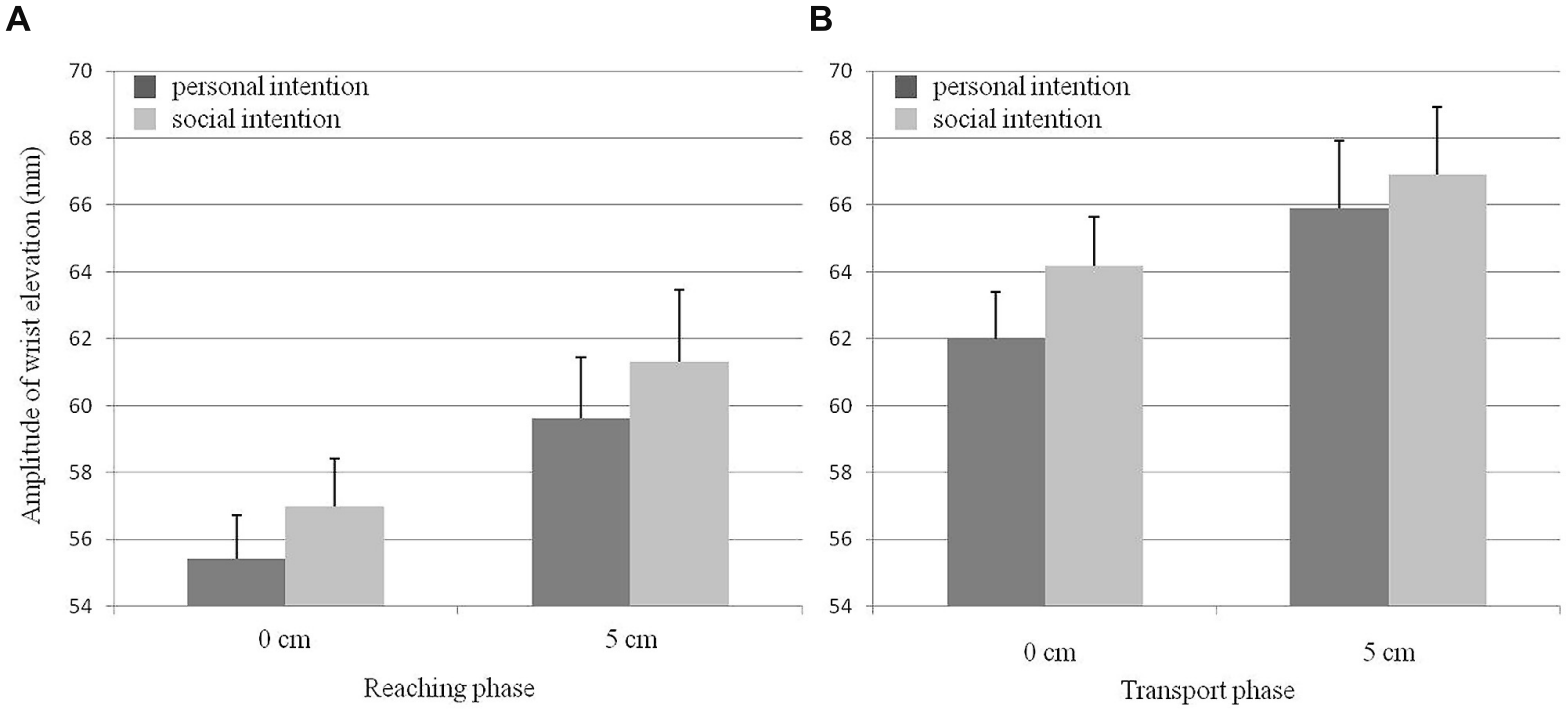
**Wrist elevation as a function of social intention and partner’s eye level for both (A) the reaching and (B) the transport phases of the *Preparatory action***. Error bars represent the SE.

Wrist elevation was influenced by social intention [*F*(1,20) = 8.01, *p* < 0.01, $\eta_{p}^{2}$ = 0.29], with a higher trajectory when participants endorsed a social (62.3 mm) rather than a personal (60.7 mm) intention. Wrist elevation was also influenced by the movement phase [*F*(1,20) = 73, *p* < 0.001, $\eta_{p}^{2}$ = 0.78], with a higher trajectory during the transport (64.3 mm) than the grasping (58.3 mm) phase. Finally, wrist elevation was influenced by eye level [*F*(1,20) = 5.3, *p* < 0.05, $\eta_{p}^{2}$ = 0.21], with a higher trajectory when participants were in the presence of a partner who had a higher seat (5 cm condition, 63.4 mm) than a seat at the same height as theirs (0 cm condition, 59.6 mm). However, there was no interaction between the three factors [social intention/movement phase: *F*(1,20) = 0.01, social intention/eye level: *F*(1,20) = 0.29, movement phase/eye level: *F*(1,20) = 0.87, social intention/movement phase/eye level: *F*(1,20) = 1.17, all ns; see **Figure 4**].

### PEAK WRIST VELOCITY

Peak wrist velocity was not influenced by social intention [*F*(1,20) = 0.39, ns], nor by eye level [*F*(1,20) = 0.88, ns]. However, it was influenced by the movement phase [*F*(1,20) = 34, *p* < 0.001, $\eta_{p}^{2}$ = 0.63], with a lower peak wrist velocity during the transport (522 mm s^-1^) than the grasping (607 mm s^-1^) phase, although more when endorsing a social (-101 mm s^-1^) rather than a personal (-70 mm s^-1^) intention, as shown by the significant intention/movement phase interaction [*F*(1,20) = 24.4, *p*<0.001, $\eta_{p}^{2}$ = 0.55]. All the other interactions were not significant [social intention/eye level: *F*(1,20) = 0.12, movement phase/eye level: *F*(1,20) = 1.37, social intention/movement phase/eye level: *F*(1,20) = 0.86, all ns].

## DISCUSSION

In the present study, we examined the role of the eye level of a confederate in the execution of individual or cooperative voluntary reach-to-grasp movements. First of all, our data confirm previous findings concerning the effect on an actor of endorsing social intention. Analyses of the *Preparatory action* revealed that participants took more time to initiate their action, which was performed at a slower speed and with a higher hand trajectory, when they placed the wooden dowel knowing that the *Main action* would be performed by the partner (Becchio et al., 2008b; Quesque et al., 2013). It is also worth noting that, although spatio-temporal variations relating to social intention were quite subtle (around 80 ms for reaction time, 20 ms for movement duration and 2 mm for wrist elevation), they were significant and consistent across different studies (Quesque et al., 2013). Taken together, these effects support the hypothesis that when endorsing a social intention, humans tend to exaggerate the spatio-temporal parameters of their movements, probably in order to facilitate the confederate’s detection of the motor and social goals of the planned action, and thus improve cooperative situations. This interpretation is supported by the findings showing that humans tend to increase the amplitude of their actions when performing explicit intentional communicative (pantomime) compared to non-communicative (actual use) object-related movements (Hermsdörfer et al., 2006, 2012).

The novelty of this study is that the unnoticed modification of the body characteristics of a partner had a sharp effect on the spatio-temporal parameters of object-oriented voluntary action. In particular, modifying the partner’s eye level had an effect on the *Preparatory action* with participants producing movements with a higher amplitude when the partner’s seat was 5 cm higher than when it was at the same height as their own, suggesting that the properties of the other person’s body are implicitly taken into account when producing a motor action in a social context. These modulations of hand trajectory when acting in a social context might reflect specific attention allocation to several sources of information, as requested by the task (Howard and Tipper, 1997; Diedrichsen et al., 2004). For example, one may speculate that when a person performs a voluntary motor action in the presence of a partner, the latter’s eye level represents a spatial target influencing the movement parameters specified to reach a particular object in the environment. The fact that social context influences object-oriented motor actions has already been suggested in previous studies (e.g., Cleret de Langavant et al., 2011; Gianelli et al., 2011; Quesque et al., 2013). In particular, Cleret de Langavant et al. (2011) showed that trajectories of pointing movements performed after giving a verbal instruction to a confederate slightly shifted in the direction of the confederate compared to pointing movements performed in a non-communicative context. The new result here is that the height of the confederate is also considered when planning and executing object-oriented motor actions. Though the present study focuses on eye level, it is worth noting that other cues might have contributed to the observed effect, as for instance body, shoulder or head height, the bending of the head or even a change in arm posture. However, previous works have shown that gaze is a crucial component of social interactions (Argyle and Cook, 1976; Kleinke, 1986; Langton et al., 2000; Becchio et al., 2008a) and that gaze direction influences motor kinematics in a social context (Ferri et al., 2011; Innocenti et al., 2012; Scorolli et al., 2014). No previous study has shown that body height in itself or a change in arm posture influences motor kinematics in a social context. Taken together, these data suggest that eye level contributes to the social effect observed in the present study, though whether other information contributes to the effect remains an open question.

Strikingly, the effect of the partner’s eye level appeared both when social intention was endorsed and when participants followed personal goals. In fact, no interaction was found between the factors Social intention and Partner’s eye level, suggesting that even in the absence of communicative instructions, the gaze characteristics of a conspecific are taken into account when planning an object-oriented motor action. These results confirm the special importance of human bodies in motor performances in a social context (Cleret de Langavant et al., 2012). They also corroborate previous findings that the presence of conspecifics automatically leads to considering their perspectives (Mainwaring et al., 2003; Tversky and Martin Hard, 2009; Qureshi et al., 2010; Samson et al., 2010) and to processing objects in the environment with reference to them (Becchio et al., 2011). It remains possible that changing the eye level of the partner influenced the head-eye coordination strategies of participants, resulting in a change in movement control. However, although head-eye movements were not recorded, this could hardly account for the observed effect of the partner’s eye level on movement amplitude since the participants and the partner had to keep their gaze on the wooden dowel when either was acting. It is worth noting that, in our study, the interactions between participants and the partner occurred in a pre-specified cooperative context. It thus remains to be established whether the influence of conspecific gaze characteristics on motor performances is still effective when the conspecifics are no longer partners but competitors. It would also be interesting to evaluate in future research whether the influence of body characteristics of conspecifics arises in a non-predefined communicative context and in a multi-agent social context.

In conclusion, although further investigations are necessary to unravel the effect of social intention on voluntary motor action, the present study demonstrates that the body characteristics of a partner, in particular their eye level, are implicitly taken into account when performing a motor action in cooperative and non-cooperative tasks. This suggests that the conspecific’s body represents one of the crucial variables that constrain motor planning and execution.

## Conflict of Interest Statement

The authors declare that the research was conducted in the absence of any commercial or financial relationships that could be construed as a potential conflict of interest.
